# Pandoomain, a scalable pipeline for genomic and protein domain context analysis, reveals widespread PT-TG domain architectural diversity and novel polymorphic toxins

**DOI:** 10.1128/msystems.00427-26

**Published:** 2026-07-30

**Authors:** Emanuel Becerra Soto, Adam J. Oliver, Marcos H. de Moraes

**Affiliations:** 1 Department of BioSciences, Rice University 124574, Houston, Texas, USA; 2 Systems, Synthetic, and Physical Biology Graduate Program, Rice University 3990, Houston, Texas, USA; University of Minnesota Twin Cities, Minneapolis, Minnesota, USA; Newcastle University Institute for Cell and Molecular Biosciences, Dundee, United Kingdom; Pacific Northwest National Laboratory, Richland, Washington, USA

**Keywords:** genome analysis, protein function, toxins

## Abstract

The rapid expansion of bacterial genome databases presents significant opportunities for functional discovery, as a large fraction of genes and protein domains remain uncharacterized. Analyzing genomic context and domain architecture is a powerful approach for functional inference, but existing tools often lack the scalability and integrated workflow required for high-throughput analysis. To address this, we developed Pandoomain, a Snakemake pipeline that automates the acquisition of genomes from the National Center for Biotechnology Information, identifies proteins of interest using hidden Markov models (HMMs), and performs systematic domain annotation and gene neighborhood analysis. We demonstrate the utility of Pandoomain through a comprehensive analysis of the poorly characterized pre-toxin TG (PT-TG) domain across 347,289 bacterial genomes. Our analysis revealed 10,226 PT-TG-containing proteins organized into 312 unique domain architectures, highlighting their association with diverse interbacterial antagonistic systems, including the Type VI secretion, Type VII secretion, and contact-dependent inhibition systems. By leveraging genomic context, we identified a novel variant of the WXG trafficking domain, termed W10XG, and subsequently discovered 24 new families of associated toxin domains. We experimentally validated six of these toxins, confirming that all six are neutralized by their cognate immunity proteins. Pandoomain is an accessible tool that enables systematic, large-scale exploration of protein domains, and our analysis of the PT-TG domain provides a rich resource for future investigations into the mechanisms and evolution of bacterial antagonism.

**IMPORTANCE:**

The rapid growth of bacterial genomic data presents a major hurdle for scientists seeking to understand the functions of newly discovered genes and proteins. To address this issue, we created Pandoomain, a powerful, accessible software tool that automates large-scale analysis of genetic information across hundreds of thousands of genomes. Using Pandoomain, we investigated a poorly understood family of proteins involved in bacterial competition, revealing novel protein domain architectural diversity. This led to the discovery of 24 new families of toxins predicted to be used by bacteria to attack their competitors, and we experimentally confirmed the toxic activity of six of them. Our work provides the scientific community with a robust tool to accelerate functional discovery and offers new insights into the evolution of bacterial conflicts, which may provide insights into the compositional dynamics of microbial communities and support methods to engineer their composition.

## INTRODUCTION

The continued expansion of public repositories, which now host millions of bacterial genomes originated from pure cultures and microbiomes, presents both a significant opportunity for discovery and a substantial analytical challenge. Often, this expansion has not been followed by elucidation of biological functions, as 30% to 40% of bacterial genes and many protein domains remain uncharacterized (1–3). To address this challenge, one powerful approach is the analysis of genomic context. This method relies on the principle that genes with related functions are often physically clustered on the chromosome in conserved gene neighborhoods or operons (4). This genomic co-localization is a strong evolutionary signature of a functional linkage and has been instrumental in revealing the roles of novel proteins within complex biological processes, including metabolic pathways and protein secretion systems. A complementary approach is the analysis of protein domain architecture, the specific composition and organization of functional domains within a protein, which provides direct clues to its function.

Investigating gene and protein domain co-localization has been extremely successful in the discovery of novel functions of antimicrobial bacterial toxin domains and their biological functions. Previous foundational work identified dozens of novel toxins and trafficking domains predicted to be involved in interbacterial antagonism (5, 6). Subsequent studies of new bacterial toxins through genomic analysis continue to yield valuable insights and offer comprehensive databases for the scientific community to explore in functional analysis (7–10). Furthermore, this context-driven approach is broadly applicable to the discovery of novel biological functions beyond interbacterial mechanisms. Examples of these novel functions include viral defense mechanisms, signal transduction cascades, and biosynthetic pathways (11–15).

A variety of bioinformatic tools exist to facilitate the analysis of protein domain architecture and genomic context. While the examples discussed here are representative of a larger landscape of available options, many of which are highly specialized for niche applications, they can be broadly categorized as either web-based servers or command-line pipelines. Web-based platforms such as the Kyoto Encyclopedia of Genes and Genomes (16), BioCyc (17), InterPro (18) web interface, the EFI-Genome Neighborhood Tool (19), and BRENDA (20) offer user-friendly graphical interfaces ideal for small-scale visualization but lack the scalability and customizability required for analyzing thousands of genomes. In contrast, command-line pipelines like Bactopia (21), ProkFunFind (22), and Domainator (23) provide the power and flexibility for high-throughput analysis, though they typically require significant bioinformatics expertise for setup and may not be able to be executed locally for vast data sets. A significant gap, therefore, exists for a tool that combines the scalability of command-line approaches with an accessible, automated workflow, as many existing solutions lack a cohesive, end-to-end framework that integrates data acquisition, taxonomic information, and systematic architecture comparison into a single, reproducible pipeline.

**Fig 1:**
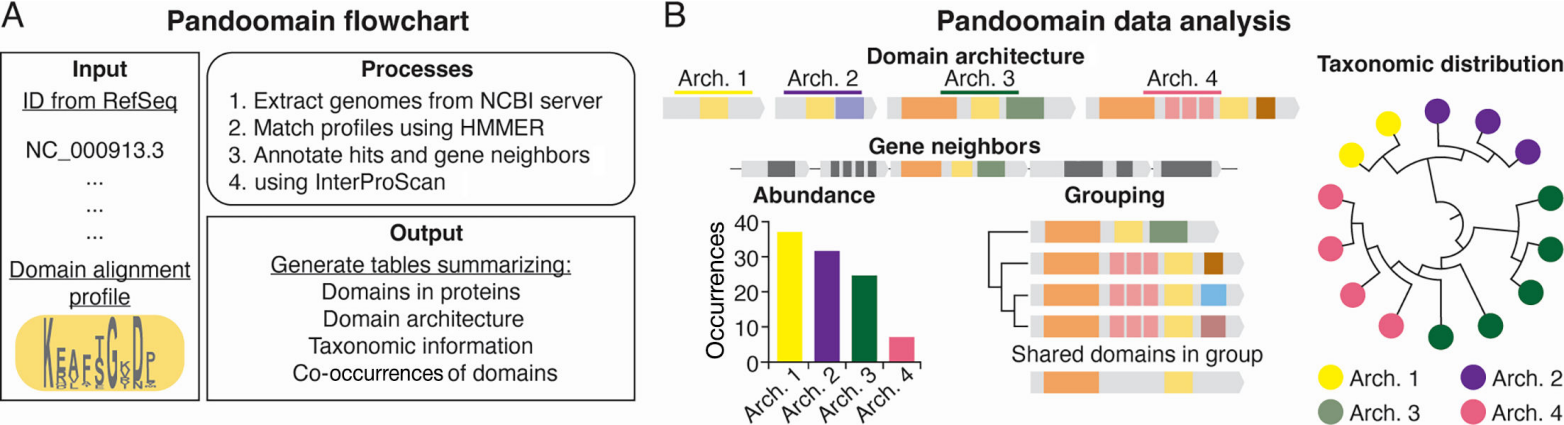
Overview of the Pandoomain workflow and data analysis pipeline. (**A**) The flowchart illustrates the main operational steps of Pandoomain. (**B**) Visualization of the output depicting domain architecture, gene neighborhood, the relative abundance of each architecture, the grouping of similar architectures based on shared domains, and their taxonomic distribution.

To address these limitations, we developed Pandoomain, an automated and reproducible pipeline focused on analyzing domain architecture and genomic context across large numbers of genomes extracted from the National Center for Biotechnology Information (NCBI). In this work, we describe the Pandoomain pipeline and validate its utility through a comprehensive analysis of the PT-TG domain. We chose this domain due to its poor characterization and its widespread distribution in polymorphic toxin systems (24, 25) (Fig. 1). This analysis serves not only to demonstrate the power of our tool but also to uncover novel features of the PT-TG domain family and its role in interbacterial antagonism. By releasing Pandoomain and this comprehensive data set as open-access resources, we equip researchers with a highly versatile pipeline to map genetic neighborhoods and illuminate uncharacterized biological functions across microbial organisms.

## MATERIALS AND METHODS

### Pandoomain implementation and workflow

The Pandoomain workflow is managed by a Snakemake (26) pipeline that executes a series of modular scripts, with key parameters specified in a config.yaml file. The pipeline is initiated with a user-supplied list of NCBI RefSeq (27) genome assembly accessions. Using this list, Pandoomain automates the download of the corresponding protein FASTA file and general feature file (GFF) for each genome from the NCBI Assembly database, storing them locally for subsequent analysis. Pandoomain downloads this pre-compiled database and merges it with the genome metadata, producing a file (genomes_ranks.tsv) with standardized and complete taxonomic information for the ranks superkingdom, phylum, class, order, family, genus, and species.

Once the genomic data are acquired, Pandoomain uses the HMMER homology search algorithm (28) to scan all downloaded proteomes against a user-provided directory of hidden Markov model (HMM) profiles. The HMM profiles can be generated by the user with the HMMER package or downloaded from different databases. This step identifies all instances of the target protein families of interest, producing a detailed report (hmmer.tsv) of all hits, their genomic locations, and search scores.

Following the identification of target proteins, Pandoomain systematically analyzes the genomic neighborhood surrounding each hit. The size of this neighborhood is a user-configurable parameter, defaulting to 12 genes upstream and downstream. This analysis yields a catalog (neighbors.tsv) with every protein within these defined neighborhoods.

The pipeline then extracts the amino acid sequences of all identified proteins, both the initial HMMER hits and their neighbors, into a single multi-FASTA file (all.faa). This file is then processed in batches by InterProScan (18), a powerful tool for large-scale protein domain annotation.

The script processes the InterProScan output to define the domain architecture of each protein. Each identified domain is assigned a random character for downstream similarity analysis between domain architectures and to facilitate inspection by the user. Subsequently, the script synthesizes the taxonomic data, protein hit information, and domain annotations to generate a presence-absence matrix (absence_presence.tsv). This matrix provides a clear, high-level overview of the distribution of every identified protein domain across all input genomes, a format ideally suited for downstream phylogenetic profiling, co-evolutionary analysis, and statistical comparisons.

### Defining domain architecture groups

To categorize diverse domain architectures, we transformed each protein into a string-based representation, assigning each unique Pfam ID a single-character “word.” This allowed us to represent the domain architecture of each protein as a “sentence” of characters. We then calculated a similarity matrix for all protein domain architecture pairs using the Jaccard index (29), which measures the ratio of shared domains (intersection) over the total unique domains present in both proteins (union).

Using the R package ape, we generated a similarity dendrogram via UPGMA clustering. The resulting tree was visualized and manually partitioned into major architectural clades using the iTOL web interface (30).

### Taxonomy analysis and visualization

To visualize the taxonomic distribution of the identified architecture groups, we used the Environment for Tree Exploration Python toolkit (31). Specifically, the ncbi_taxonomy module was used to build a phylogenetic tree based on the NCBI taxonomy IDs obtained from each genome’s metadata. The final tree, annotated with the domain architecture groups, was generated using the iTOL web interface.

### Identification of new W10XG toxins

To identify new W10XG toxins, proteins containing W10XG domains were filtered to remove sequences with fewer than 260 amino acids. This threshold was defined because it is in this approximate region that the PT-TG domain ends and the toxin domain starts. The putative toxin domains were extracted from the C-terminal portion of the proteins, from position 260 to the end, and aligned using MAFFT (32). The toxins were then clustered into homology groups sharing more than 70% sequence identity. One random member of each group was selected for further analysis. Putative immunity proteins were identified from the genes immediately downstream of the toxin gene. The structure of the toxin and immunity multimer was modeled using the AlphaFold web server (33). Resulting protein structures were individually curated, and toxin-immunity pairs where the toxin domain appeared truncated, or there was no predicted interaction between toxin and immunity, were excluded. Finally, the resulting toxin domains were aligned using the FoldMason tool available on Foldseek web server (34). If structural homologs were identified, only one was selected for further analysis.

### Bacterial culture methods and plasmid construction

*Escherichia coli* DH5α strains used in this study were cultivated in Lysogeny Broth (LB) at 37°C with shaking or on LB medium solidified with 1.5% agar. When necessary, antibiotics were supplied to the media in the following concentrations: gentamicin (15 μg/m^L−1^) and trimethoprim (50 μg/m^L−1^). Synthetic DNA fragments containing codon-optimized toxin and immunity protein genes were acquired from Twist Bioscience (Table 1). The fragments were designed with terminal homology regions for cloning into expression vectors via ClonExpress Ultra One Step Cloning Kit V3 (Vazyme). Toxin genes were cloned into the rhamnose-inducible vector pSCRhaB2 (35), and cognate immunity genes were cloned into the isopropyl β-D-1-thiogalactopyranoside (IPTG) inducible vector pPSV39 (36). To prevent self-intoxication during the cloning process, toxin constructs were transformed into *E. coli* DH5α already carrying the plasmid for the corresponding immunity protein.

**TABLE 1:** Sequence of synthetic DNA fragments used for cloning and expression of toxin-immunity pairs

Construct	Sequence
Tox3^Pt^	AATGAAATTCAGCAGGATCACATATGCCATGGGTACTGGAAAAACGGTGAA CGATTTAATACATGATCCGAAGCTGAAGAATTTAATGCAAGACGCGAAATTG ATTAAGGATAAAGGCCGCGCCACAATTTACGACAAAACGGGCGGATACAAT CAAGCCGTTAAGGAATTCGATGAACTCAATCCGAAAAATATTAAACAACTGG TAGACAAAAAGACAGGAGACGTCATTGGAAAGCAGGGCCAGATAACGCTC GATGGGAAAACAGTTAATATAAACGTTCGCAAAATAAGTTCTCATGATAATCG CCCTACTATTGAGATTATAATCGACAAATATGACCGCATCAAATTTAGATATTA ATCTAGAGTCGACCTGCAGGCATGCA
Imm3^Pt^	GGATAACAATTTCAGAATTCGAGCTCACGGGAGGAAAGATGGAGAAGGAA C GCTGGGATAAATGGCAGCCTAAAATTGAATTTCCCAACAAGATCTGGTTCGA GAGCCTTTTCCAAGACCGCTCTGGCCTGAAAATCCAGTTTGAGTCTGAAGA TAATCGTAAAGTAATTGTAATCTTTGAACATTCTGTGCTTAGTTACCGGGTAAC TGATGAAGGTGATCTGTTGCAGACCATCTCGTTTTGGTCTAGTGAATATGGTA ATGATTTTTTCAGTTGGCCGCTGTATAAATCAAGCAATTCATCATTTATCAATTG GTTCAATGAAGAAAGTTGCGGTAAGTTTGAAGAAGAACACATCGAACATTATG TTTTTATAACCCCTAATGAAATTATCGATGTATTGTCCGCAATACCACCGCAATT AATCATCTCTAACTAATCTAGATATACAGATATTGAAATGAATAGA
Tox7^Ch^	AATGAAATTCAGCAGGATCACATATGCCATGAGCAACGCCTTCCAGTGGACG GGTAAAAACTTTTCTCAGATGGGAAAAAATATGTCCCCGAACGAAATGATAAG CAACTTAGAAAAGTCAGGCTGGAATAAGATCGTGGAACCGGGGGGGAAAAA ATCTGGTCCAGCAACAATCTTCACGGACCCGTCAACGGGCACCAAGGTACG AATCCATGCCGAACCAGGTGAAGGCACACCGTATTTTCGTGTACAGAATAAA GGTGGTGGGTATCTGGATAACAATGGTGTCTTCCCTAGCAATGCGACGAAAC AAGAACTGCGCGACAAAACCCATTTCTATTTCGAAAAGTAATCTAGAGTCGAC CTGCAGGCATGCA
Imm7^Ch^	GGATAACAATTTCAGAATTCGAGCTCACGGGAGGAAAGATGGAACTGCTTGA CGAAATTGAGAAAAAAGTTCAACGCGGCAAGTTCCTTCTTATGGTAGAAATTC TTGAAGGTTATCGGAGCAACGTGCACCAAGCGGTAAACCGGATAGACGGATC GCTGCAAATGTATCGCTCGGCTGACTCTTCCTATGCCAAACATTGGGAAGGCG AAACCCGTAACAAATATGAGGAAATTACGGCGGAGATCCAGCACATCGGTAAT AAAATAGATCAACAGGGAGACCGTTTGATAAATGCGATTAACAAAGAAATCCGG AGCCTGCTGGCGAAATGCGAGGCTTTACGGTAATCTAGATATACAGATATTGAA ATGAATAGA
Tox9^Pt^	AATGAAATTCAGCAGGATCACATATGCCATGGCCCGCCCCAGTTGGAGACAG TCTGAAATCGACATTGGCAAAGAGTACCCGGAATATTCTACTCAAAAAAGTTTC AAGAATGGTAAAGAAGTGCCTTATGGTACCAAAGGTTCCTCACGCCCTGATTA CTATAAAAAGGGCTTTTCTATCGAAGTTAAGAACTACAAAATCACCACACCGGA GGGCCGTTCTCGCCTCGTCAATAACATATCTACACAGTTTGAAAAACGTCTGA GCGATCTCCCTCGTGGCACGAAACAAACCGTAATCATTGACATTCGCGGGCA GAACGTTTCCGAGGAAATTCTTGATGATCTGTACGATAAAATCATGAAAAAAAC GAACGGCAAAGTGAACATACGCTTTAAAACAGATGAATAATCTAGAGTCGACCT GCAGGCATGCA
Imm9^Pt^	GGATAACAATTTCAGAATTCGAGCTCACGGGAGGAAAGATGGCTGTAGGGTTT AAAGTGAAGTATTATTGGTACCCAATTGGTCACGGTGATTTCTTGCATAGTTTTT TTAGTACAGTTTCATATCACTTAGAACCGAACGGTTGGGGAACTAAATATCCCCT GTTGCTGAACAAACTGTATAATGGCAAACTGGAATATAAGTATATTAAAGATGCA ATAAAGGAGATCGATGAAATACAAGAATCTCTGAAAAACTACTCCCCATCACAA GTGATCTGGGATATTGAAGATTTGTCCAAAACCCCGCCTTGGGGAGATAATATT TCTGATGAAATTACCGATCTCTCTAATTACTTCGTAACGTCAGATGGAGAAGATC TGATAACCATTCTCAAAAAGGCGCTTAACAAGGCACTGAAAGTAAAATCCGACA TTGAGATTGAAAGTATCTAATCTAGATATACAGATATTGAAATGAATAGA
Tox14^Pt^	AATGAAATTCAGCAGGATCACATATGCCATGAATTTTGTGAAGGTTGATAAAAA CAAAATCATTCAGACTGCTACTGCGCATAAGAAAGGGGGAGAAACCGTCGTCG GCCACGCGTTGCAAAAACACGCCGGTCGAAACCCACATATTTGGGGTAAAGTA AAAGGCGGCCCTGCGCAGATTAACCAAATGGCCCTGAAACACCTCCAAGAAAT CCTTAACGGTACAGGCGGATTTAAGAGAGTGAAGAATTCAAGAGGAATAGAATT CCTTGAAAAAAAATTACCGGATGGTCGGGGGGTTCGTCTGAATCTGGACGGTA CATTTAAAGGCTTTATTGATCAGTAATCTAGAGTCGACCTGCAGGCATGCA
Imm14^Pt^	GGATAACAATTTCAGAATTCGAGCTCACGGGAGGAAAGATGGAGAATTTAGAA ATCATCCTGCTTGATTTCCGTAAAGAAGAGATAGAAACTCTGATTCACGAAGAA CTGAAATTATCTACTTTAAACATTAAGAGTTCGCACTTCTATGATTTTAACAGTGG CAAGGATATGGAATTTCCGCAGGTGAAGAATATGAAAGAAATTCTTTCACCTAA AGGCACCGGTAATATTGTTTTAGAACAGCTGCAGTTGGGTATCACCCTTAAAAA CGTTGTTATTGTGTTTTCCTTTGACGAAGAATGTGGAGACATAGTTTTTAATTTT CCTGAATCAGAAATATTTATAGGTGATAAAAAAGAAGTGAAGTCACGCTTCGAA AAACTGGTTAATTATCTCGTGAAACTTAAAATGAAATATAATATCCCGAAAGTAAT TATTGGGTATGAACCAGCAGATGATGAAGACACTATGCTTATTGAACTCTCGGA CAATGCTTTGAATCTCGAGAAAGTTCTCGAAAAAATTCTTGATTAATCTAGATATA CAGATATTGAAATGAATAGA
Tox14^Pg^	AATGAAATTCAGCAGGATCACATATGCCATGAAAGGAACCGGTAACGTTGCACA CAATGCAGCACAGTATGCAAAATTAAAAACCCATTATAAGCAGTGGGAGAAATAT GGGAAAGCCGGTATTCGCGAGCTCCCAGATGGCCGTATCCGATACTATGGTAA GATAACGCCGGCCTCTAAACCGGGGGAGATGGCAGGCCGGCGCGTGGTTCG CGAGTGGAATCCAACCACTGGTCAGAAACGGACCTGGCACGAAACAGTGGAC CACAACGGGCGGATAAGACAGGTACGCCCGGATACCAAGTTCACCGGCGGTG TGAAGGTTCACTATCGATTTGATTCAAATGGAAATTATATCGGTAAATGGTAATCT AGAGTCGACCTGCAGGCATGCA
Imm14^Pg^	GGATAACAATTTCAGAATTCGAGCTCACGGGAGGAAAGATGCGTCAGCCTACA AAAAAAGAAATCATCGACAAACTGAAAATGATTTTACAGGGTAAACTCACCCGA GAAGAAGTTGCGGATTGGGCATCTGAATATGTAATGCAAGATGAGCCCAACATT TCCGATGAAACAGTTTGGGAGTTACTGCAAATCGTGAGCGGTATAGATCTGAAA GATTCACCGGATGAGTATCTGCATGTAGAACAGGATATAATCGACTGGATTAACA AATATTCTAATTAATCTAGATATACAGATATTGAAATGAATAGA
Tox23^Fo^	AATGAAATTCAGCAGGATCACATATGCCATGAATGAAGATTACTTCATCGGAACG TTAAAAGGTGAAAAAGTACATCTGAAAGGCGTTAAGGTTGAGGAGATTATATATA CCAAAAGACTTCCTGATGAAACCGCCAAATTAAGACGTGAATTTAATTCATCAGT GCGGAAAAATTTTCTGAAAGAATTTGCTAACGATCCACAACGTGTCGAATATCTG CGTACGGCTGGTCTGTCTGATGCTGATATCGCACGCATGAAAGACGGCCTGAA CCCAAAAGGTTGGCAAGTGCATCATAATCTCCCGCTTGATGACGGTGGCACGA ATGATTTTACCAACCTGGTGTTAATTAAAAATGACCCGTACCATAAAGCGATTACC AACGAACAGAACAGTTTGACTAAAGGTCTCCTCCCGAAGCAATCTAAAACAATAA ACTGGCCCTTGTTCGAAGAAGACATCTATCCGAGTAAACCATTCAAATAATCTAG AGTCGACCTGCAGGCATGCA
Imm23^Fo^	GGATAACAATTTCAGAATTCGAGCTCACGGGAGGAAAGATGTCCCAATGGATCG ATTTATTAAAAGAACTGGCTAAAATTGAGGAAAAGTATGGTGGTACCCTCAGAAA CCCCGCGTCAGAGGAAGAGATAATTAAAATGAAAAACGAAGTCAACCAGAAGTT TGAGAACGTTGTCTTACCGGACTCTTACGTTAATTTCTTAAAGGAGATCAACGGT CTTGATTTCAACGGATTAGTCTTTTATGGCGTGGATGAGCGATTGCTGGAGCAG GACGTGGAAGAGGTTCACGGCTTTATAGAAACGAACGAGCTTTGGTATGAAAAC GATTGGCAAAGACAGTACATTTTCTTTGGCGACTCGGACACAGCATGGTATTGC TATAACAGCAAAGAAAACAACTATGTTGAGCTTGATAAGCCGAGTGGAACGCTGA TCCAATCGTTTGAGTCATTCGATTCTATGCTGTATGATGCTCTTAAAACCGTGCTG TTATAATCTAGATATACAGATATTGAAATGAATAGA

### Validation of W10XG toxins

To test the activity of the toxin-immunity pairs, overnight cultures of freshly picked colonies of *E. coli* carrying the dual-plasmid toxin and immunity protein expression system were diluted 1:100 into fresh LB medium. The cultures were grown with shaking at 37°C to an OD_600_ of approximately 0.6. Expression was then induced under three conditions: toxin expression was induced with 3 mM rhamnose, co-expression of the toxin and immunity protein was induced with 3 mM rhamnose, and 1 mM IPTG was added 20 min before rhamnose to enable a buildup of immunity protein. A third culture was left uninduced as a negative control. Four biological replicates were used. After a 90-min incubation period, samples were collected, serially diluted, and plated onto LB agar. Plates were incubated overnight at 37°C, and the resulting colonies were counted to determine cell viability (CFU/mL^−1^). The experiments were performed twice across 2 separate days. To identify whether the observed changes in cell viability resulted specifically from the toxin or from the toxin and immunity proteins, cultures of *E. coli* transformed with empty pSCRhaB2 and pPSV39 vectors, or only pPSV39 with the immunity genes, underwent the same toxin and immunity induction protocol as the strains carrying toxin-immunity pairs. Statistical analysis was performed with GraphPad Prism software using one-way analysis of variance and *post hoc* two-tailed Welch’s *t*-tests. *P*-values less than 0.05 were considered statistically significant.

## RESULTS

### Implementation

We opted to use a SnakeMake architecture due to its modularity, which ensures that all intermediate files, such as the HMMER results and neighbor lists, are also available for detailed inspection, offering researchers multiple avenues for hypothesis generation and follow-up analysis. SnakeMake also executes the required scripts without requiring user input, which streamlines the entire process. The RefSeq genome accessions can be easily extracted from the NCBI Datasets web page that allows the search of entries using the name of organisms or specific TaxIDs. The web page also allows filtering by assembly status, deposit year, and other parameters. Additionally, the NCBI data sets command-line tools (37) can be used to download large numbers of accessions. Pandoomain then automates the download of the protein FASTA and GFF annotation files for all the specified genomes from the NCBI Assembly database. After the conclusion of this step, all the data are locally available for subsequent, rapid processing.

The Pandoomain analysis pipeline begins by identifying proteins that match a user-provided hidden Markov model (HMM) profile. This HMM alignment can be generated by the user from a custom set of amino acid sequences or acquired from public databases. The InterPro database is a valuable source for such profiles, as it integrates data from 13 different member databases, providing a comprehensive collection of protein signatures.

The integration of taxonomic and genomic data is a common challenge in functional genomic analysis. The NCBI taxonomic data are organized in a tree topology where each taxon is assigned to up to 41 taxonomic ranks, such as class, order, and genus. While comprehensive and well-curated, entries in this database often lack taxonomic ranks, creating challenges for comparative high-throughput analysis. By employing the Taxallnomy tool (38), we can obtain a hierarchically complete lineage with all ranks of taxa available in the NCBI Taxonomy database. As a result, we can determine the taxonomic relationship between all the organisms in the analysis.

A key feature of the pipeline is the automated analysis of the genomic neighborhood surrounding each protein identified by the HMM search. The pipeline defines the neighborhood as a user-configurable number of genes flanking the target gene, typically 12 upstream and 12 downstream. All proteins within this window are then annotated using InterproScan against the Interpro database. This automated annotation of the genomic context leverages the principle of “guilt by association” to make functional inferences.

**Fig 2:**
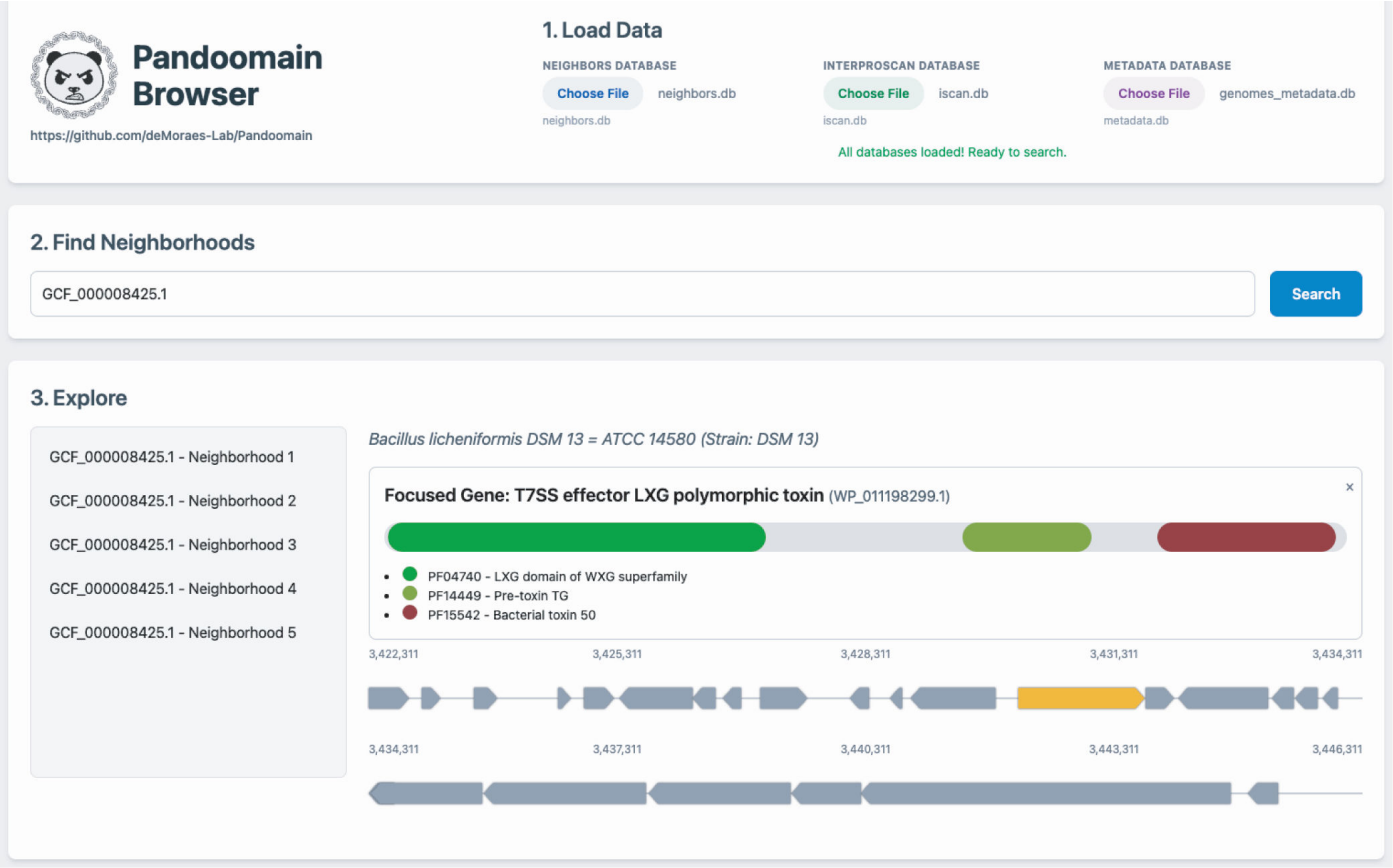
Interface of the Pandoomain Browser. The standalone HTML browser facilitates the local visualization of SQLite output files generated by the Pandoomain pipeline. Users can query specific protein or genome identifiers to explore local genomic neighborhoods, enabling the rapid identification of conserved gene associations and protein domain architectures.

The Pandoomain Browser provides a powerful resource for rapid and intuitive data exploration without the requirement for command-line proficiency. The browser is implemented as a standalone HTML file compatible with standard web browsers, which processes Pandoomain output data locally (Fig. 2). To initiate an analysis, users load three SQLite database files generated by the pipeline: neighbors.db, iscan.db, and taxonomy.db, which contain gene neighborhood architecture, protein domain annotations, and taxonomic metadata, respectively. Within the interface, users can query specific protein accessions or genomes to identify relevant hits. Selecting a hit generates a dynamic graphical depiction of the genomic neighborhood, where individual genes are represented as arrows. Furthermore, specific proteins can be expanded to visualize their constituent domains, allowing for the rapid assessment of domain architecture and potential function across large data sets (Fig. 2).

### Application of Pandoomain to categorize proteins containing PT-TG domains

Bacterial polymorphic toxins are composed of different trafficking domains associated with toxin domains. In many cases, they also contain pre-toxin domains (6). The pre-toxin TG domain (PT-TG, Pfam ID: PF14449) named for its conserved TG motif, is predicted to function as a linker between toxin and trafficking domains or to mediate interactions with the target cell envelope. This domain is associated with different antagonistic system domains, including WXG, LXG, ALF, and diverse toxins (24). To the best of our knowledge, the PT-TG domain structure remains unresolved, likely due to intrinsic disorder, and its specific cellular function is uncharacterized.

The ubiquitous nature of the PT-TG domain, coupled with its modular association across disparate antagonistic systems, led us to hypothesize that this domain functions in a broader range of biological processes than currently recognized. Pandoomain is uniquely suited to test this hypothesis, as this pipeline can identify novel architectures containing PT-TG and potential toxin-immunity pairings that remain obscured in smaller-scale analyses. To validate the applicability of Pandoomain, we proceed with an in-depth investigation of PT-TG across a large data set of bacterial genomes.

**Fig 3:**
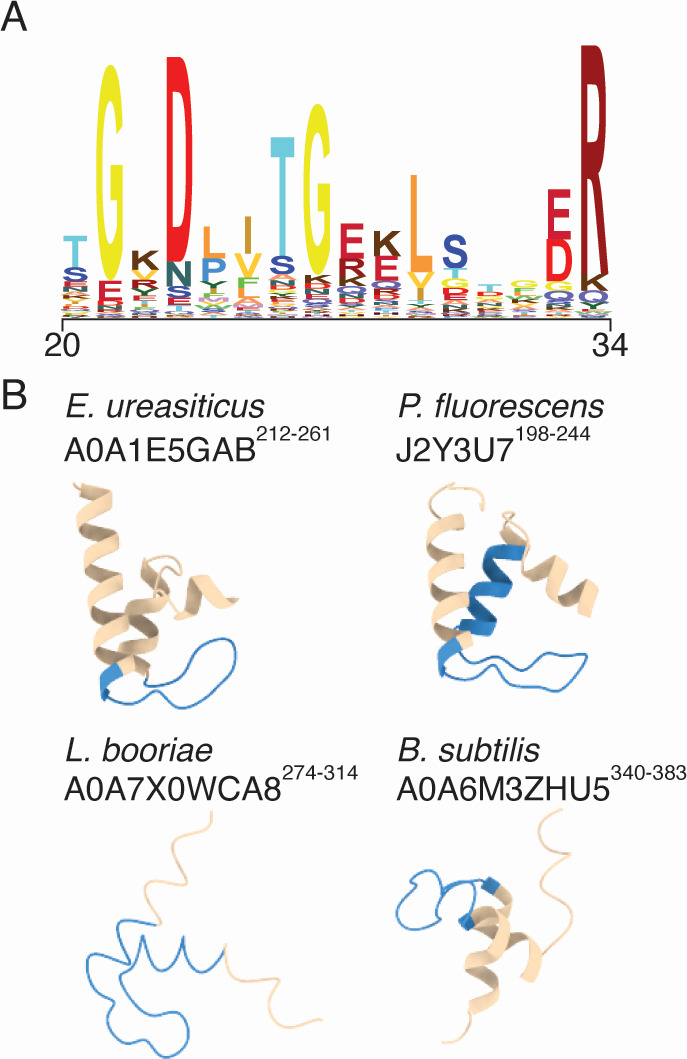
The conserved residues of PT-TG are located in an unorganized region. (**A**) A sequence logo depicting the conserved 15-amino-acid region of the PT-TG domain shows that residues including a Gly, Asp, and Arg are more conserved than the nominal TG motif. (**B**) AlphaFold3 structural models of the PT-TG domain from four different bacterial proteins originating from *Enterococcus ureasiticus*, *Pseudomonas fluorescens*, *Listeria booriae*, and *Bacillus subtilis*. The models predict that the highly conserved 15-residue region (blue) lacks secondary structure.

First, we generated an HMM alignment using the seed sequences from the PT-TG Pfam database. This analysis showed that other residues, including a Gly, Asp, and Arg within a 15 amino acid region, are more conserved than the nominal TG motif (Fig. 3A). We next used AlphaFold3 to model the structure of proteins containing a PT-TG domain. Consistent with its presumed disordered nature, this region was not predicted with high confidence (pLDDT < 70). Despite the overall low confidence, the models consistently predicted that the highly conserved 15-residue region forms a loop (Fig. 3B, the 15-residue regions are colored blue). The combination of high residue conservation and a consistent structural prediction of a loop suggests this region is a key functional element of the PT-TG domain, likely mediating molecular interactions required for toxin delivery.

### Classification of domain architectures containing PT-TG

Using an HMM query generated with the PT-TG alignment available on Interpro, we employed Pandoomain to search for homologs across ~300,000 bacterial genomes. The selected genomes were filtered based on the CheckM (39) completeness of >95% and contamination <0.5%. To reduce the presence of genomes from clonal strains, we selected only one genome per Bioproject deposition. This step reduces the amount of redundant data without the need to apply computationally intensive algorithms to identify nearly identical genomes over the >2.5M entries available on NCBI.

Using Pandoomain, we analyzed the selected genomes and identified 10,226 proteins containing the PT-TG domain distributed across 22,629 genomes, highlighting its widespread prevalence. These proteins showed remarkable architectural diversity, comprising 312 unique arrangements that combine the PT-TG domain with 111 other distinct protein domains (Tables S1 and S2).

The entire analysis was performed on a PC (i7-1165G7 processor, 16 GB RAM), with a total runtime of approximately 19 h. An external solid-state drive was necessary to store the large number of genomic files downloaded. While all local computation steps completed without issue, the initial data acquisition was occasionally interrupted. Users should be aware that the download phase is dependent on the connection stability with NCBI servers, and interruptions can be a common issue when retrieving large data sets.

To manage the complexity of 312 unique domain architectures, we clustered them into a smaller number of functional groups using the Jaccard index. We chose this metric as it provides a robust measure of similarity based on the presence or absence of unique domains, which is particularly effective for this analysis since many toxins feature repeated domains within a single protein. This approach disregards the number of repeated domains within a given protein, allowing us to group architectures that share the same core trafficking domains, even if they are associated with different toxin domains.

**Fig 4:**
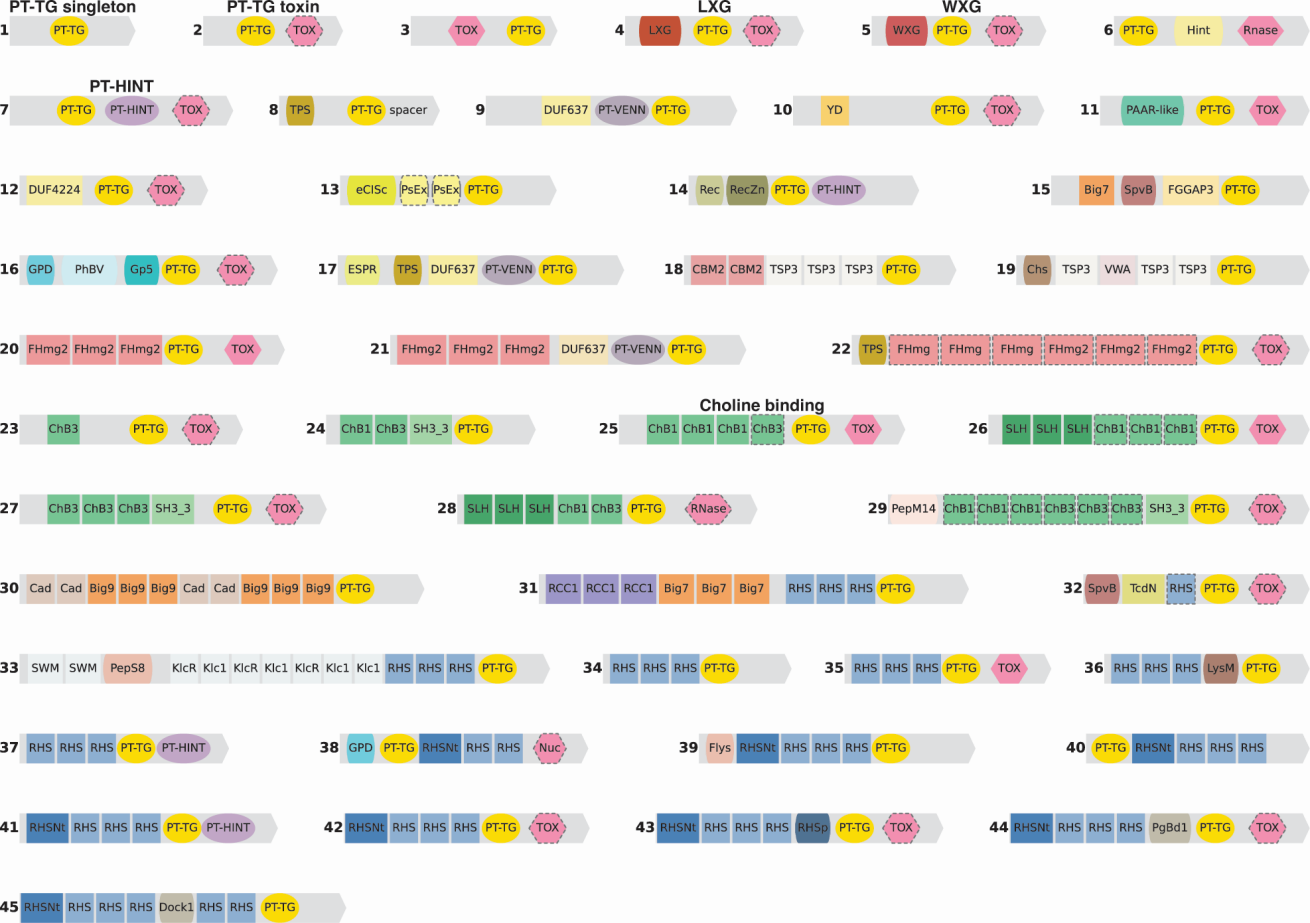
Pandoomain analysis reveals 45 major groups of PT-TG domain architectures. The 312 unique domain architectures were clustered into 45 main groups based on the Jaccard index similarity of their component domains. Each numbered bar represents the consensus architecture for a group, with distinct protein domains indicated by colored boxes. The architectures that include 95% of all proteins are named. The colored shapes indicate their function: rectangle, repetitive domain; ellipse, pre-toxin; hexagon, toxin; and rounded rectangle, other. Dotted lines indicate domains that may be absent.

This clustering approach successfully condensed the 312 domain architectures into 45 main groups (Fig. 4; Table S1), referred to as groups thereafter for simplicity. Our analysis revealed that the PT-TG domain is co-localized with trafficking domains from a wide variety of known antagonistic systems, including the VgrG from the Type VI secretion system (T6SS) (40), WXG and LXG from the Type VII secretion system (T7SS) (41, 42), and filamentous hemagglutinin (FHA)-peptide repeat from contact-dependent inhibition (CDI) systems (43). Some groups also incorporate diverse trafficking and repetitive domains, such as the choline-binding (44), cohesin (45), and dockerin (46) domains, which highlight their modular nature. A large fraction of the identified proteins lacked any recognizable toxin or other domains. This suggests that either these proteins utilize novel, uncharacterized toxin domains or they have evolved to serve functions distinct from direct interbacterial antagonism.

Notably, this analysis identified in groups 30 and 31 the presence of Bacterial Ig-like (Big) domains, PF17957 and PF17963, which were not previously associated with polymorphic toxin systems. The Big domains share a similar fold to immunoglobulins (Ig), are widespread in bacteria, and are involved in diverse roles that require interactions between molecules (47). These domains serve in different attachment roles: attaching cell surface proteins to eukaryotic host cells (48), mediating the interaction of hydrolases to polysaccharide substrates (49, 50), and supporting mating pair formation in conjugation (51). Furthermore, their presence in phages is hypothesized to contribute to adsorption (52). Moreover, in groups 30 and 31, Big domains appear in tandem repeats, suggesting they serve a structural role similar to that of RHS (53), ALF (54), and FHA (43) domains. Given that Big domains are frequently involved in cell-to-cell recognition, these domains may mediate the molecular interactions between donor and recipient cells during toxin translocation.

### Abundance and distribution of architecture groups

**Fig 5:**
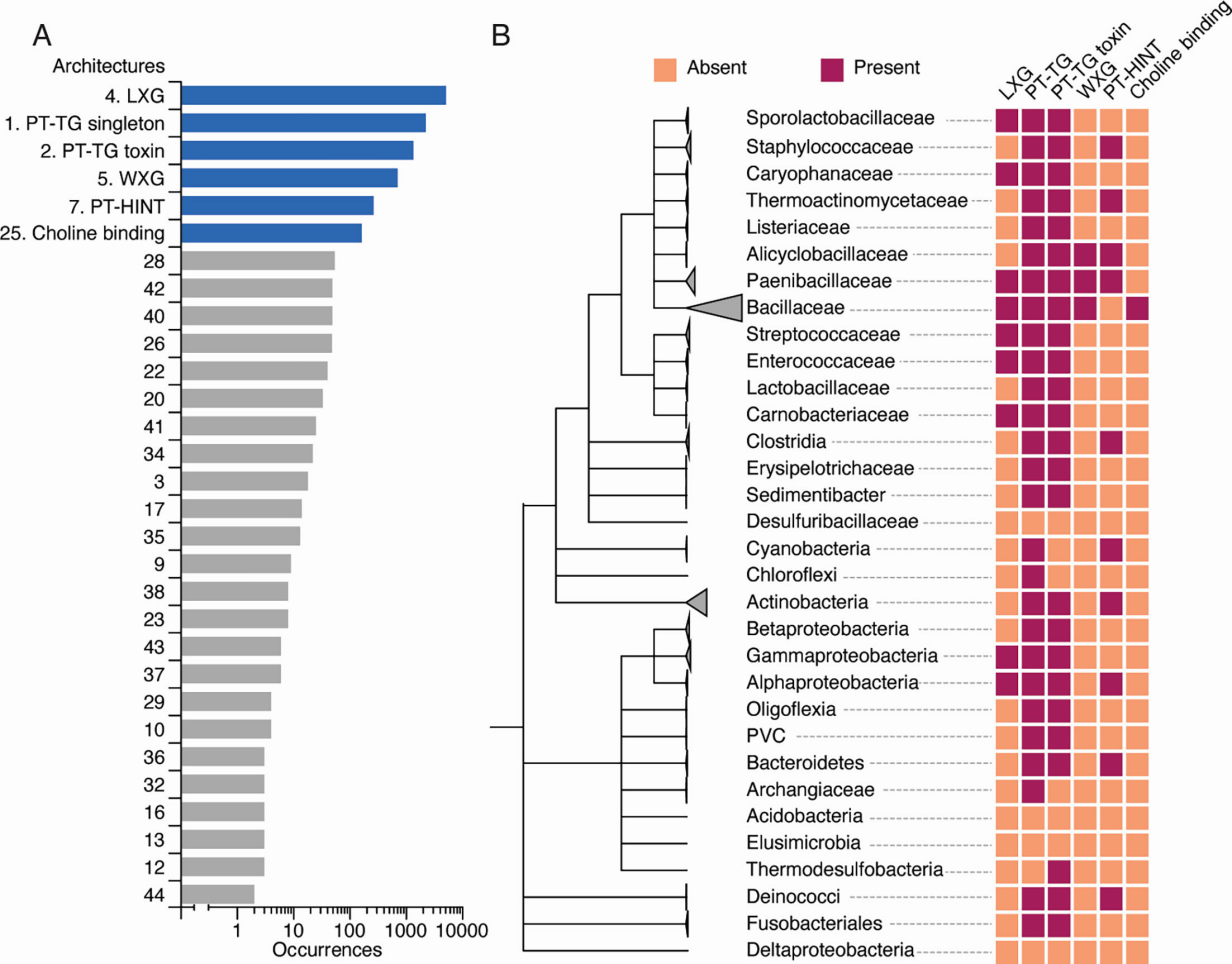
Abundance and taxonomic distribution of PT-TG domain architectures. (**A**) A bar chart showing the abundance of the PT-TG domain architecture groups with more than one member identified by Pandoomain. The top 6 most abundant groups, accounting for over 95% of all identified proteins, are highlighted in blue. (**B**) A heatmap illustrates the taxonomic distribution of the six most abundant architecture groups across a phylogenetic tree of bacterial taxa. To account for the sparse representation of certain groups, branches of the taxonomic tree are collapsed at different hierarchical levels (e.g., family, class, or phylum).

After grouping the architectures, we investigated their abundance and taxonomic distribution. The analysis revealed a highly skewed distribution. Most groups are rare, with 14 of them containing 1 member, while a few are highly abundant (Fig. 5A). Remarkably, just six groups, LXG, PT-TG singleton, PT-TG toxin, WXG, PT-HINT, and choline-binding, collectively accounted for over 95% of all identified proteins containing a PT-TG domain. Among these, the LXG and WXG groups are the only well-characterized examples, being substrates of the T7SS (41, 42).

The PT-HINT group is defined by the presence of the pre-toxin hedgehog-intein peptidase domain, which is predicted to facilitate toxin release at the cell surface (24). This group was found across many clades, primarily within the Streptomycetaceae family (Fig. 5B). We also noted that members of this group possess an unannotated N-terminal region of approximately 300 amino acids, suggesting they may utilize additional mechanisms for secretion and translocation that are unknown. Taxonomic analysis showed that proteins with a PT-TG domain are most prevalent in the family Bacillaceae (Fig. 5B). The T7SS-associated LXG and WXG domains were found almost exclusively in monoderms. The few exceptions in Gammaproteobacteria and Alphaproteobacteria were located on small contigs lacking other genes and are likely assembly artifacts. The choline-binding group was restricted to Bacillaceae, indicating it may be part of an antagonism system specific to this family. In stark contrast, the PT-TG and PT-TG toxin domain architectures were widely distributed across diverse bacterial phyla, including both monoderm and diderm lineages (Fig. 5B). Since antagonistic systems require specialized machinery to translocate toxins across distinct cell envelopes, this broad distribution suggests that these architecture groups are not a single, conserved system but are associated with multiple, distinct secretion systems or are involved in unknown cellular functions. The unexpected discovery that the PT-TG domain is associated with both monoderm and diderm bacteria opens new avenues for investigating the evolution and mechanisms of interbacterial antagonism.

### Genomic context analysis reveals a novel WXG domain variant

**Fig 6:**
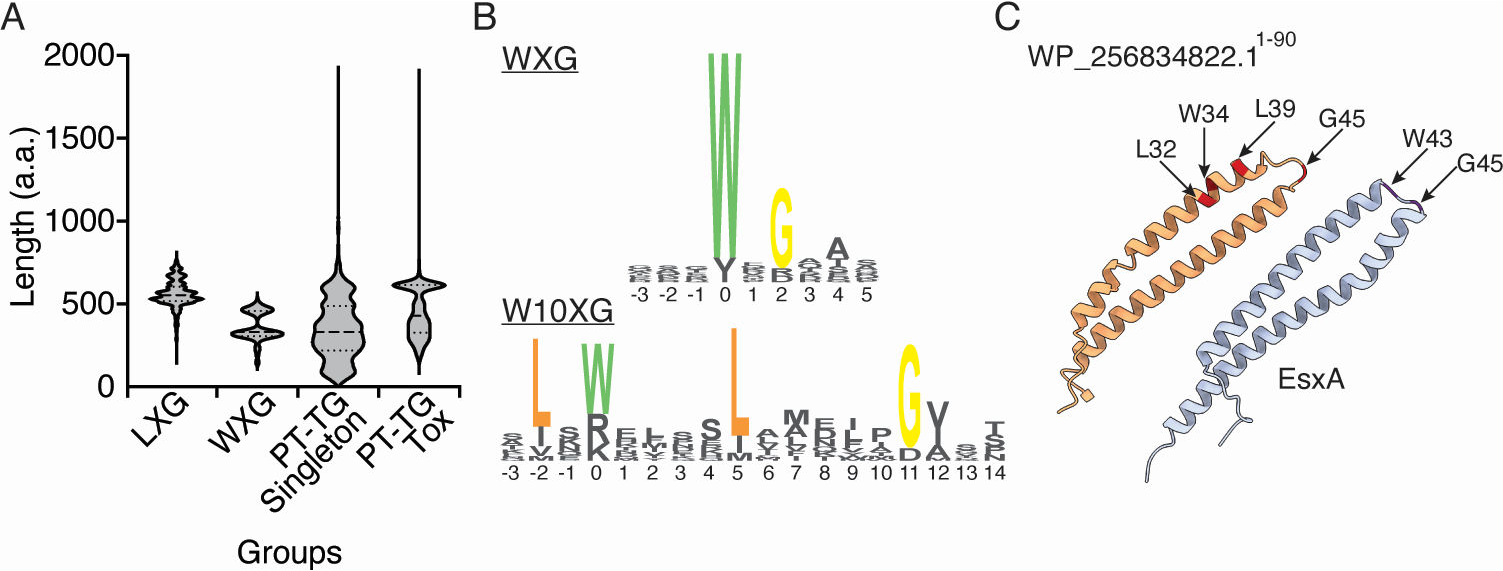
Characterization of the W10XG domain variant and its associated novel toxin families. (**A**) Violin plots comparing the amino acid (a.a.) length distributions of proteins from the LXG, WXG, PT-TG singleton, and PT-TG toxin architecture groups. The substantial size of many proteins in the PT-TG groups indicates that the absence of a known trafficking domain cannot be explained by truncation alone. (**B**) Sequence logo alignment comparing the canonical WXG domain with the W10XG variant identified in this study. The W10XG variant is defined by a characteristic 10-residue insertion between the conserved tryptophan (W) and glycine (G) residues. (**C**) Side-by-side comparison of the Alphafold3 predicted structure of the W10XG protein WP_25684822.1 from *Paenibacillus maysiensis* and the resolved structure of the WXG protein EsxA from *Staphylococcus aureus* (PDB: 2VRZ). Conserved residues are highlighted in red.

This unexpected finding that members of the PT-TG singleton and PT-TG toxin groups lack trafficking domains led us to hypothesize that these proteins could contain novel domains. To investigate this, we compared the protein length distributions of members of the PT-TG singleton and PT-TG toxin groups against those of the well-characterized LXG and WXG groups. PT-TG singleton and PT-TG toxin group members have a wider length distribution than those in the LXG and WXG groups (Fig. 6A), and many are over 250 amino acids long, surpassing the expected length for proteins containing PT-TG and toxin domains only. While these observations do not exclude the possibility that some are truncated proteins, truncation alone is unlikely to account for the general absence of a known trafficking or toxin domain.

To investigate why proteins lacked identified trafficking domains, we analyzed their genomic neighborhoods for clues to their function. We applied a statistical framework using the Apriori algorithm for association rule mining (Table S4), and we found that proteins lacking trafficking domains frequently co-occur with Type VII secretion system b (T7SSb) structural genes (55), including those encoding EsaE and WXG domains (PF16887 and PF06013 [Fig. S1]). We also identified genes encoding small α-helical proteins immediately upstream of the PT-TG genes. These proteins share structural similarities with Lap proteins, which are partners required for the secretion of LXG substrates by the T7SSb (56, 57) (Fig. S1). Collectively, our observations support the hypothesis that members of the PT-TG singleton and PT-TG toxin groups are not truncated but instead harbor trafficking domains, WXG or LXG, with homology below InterproScan’s detection threshold.

We performed a targeted HMMER search using LXG and WXG domain alignments as queries, against the PT-TG singleton and PT-TG toxin proteins found in the genomic neighborhood of T7SSb structural genes. This approach identified WXG domain homology in the N-terminal region of 112 proteins from the PT-TG singleton and 95 from the PT-TG toxin groups. The bit scores for these hits were below the Gathering Threshold defined by Pfam, explaining their initial omission. To understand the lower score, we aligned the N-terminal regions of these newly identified proteins with canonical WXG domains. The alignment revealed a distinct variation: while the canonical WXG domain contains a single amino acid between the characteristic Trp (W) and Gly (G) residues, these new proteins have a sequence of 10 amino acids with a conserved Leu (L) between the Trp and Gly residues. An additional conserved Leu was also present at the −2 position relative to the Trp (Fig. 6B). While this variant associated with the PT-TG singleton and PT-TG toxin groups does not warrant a new domain classification, for simplicity, we will refer to this group as W10XG throughout the remainder of this manuscript. To our knowledge, experimental evidence for the secretion of WXG-containing toxins by the T7SSb is currently lacking. However, the strong genomic association between these candidates and T7SSb machinery, coupled with the well-established role of WXG-family proteins (e.g., EsxA) as secreted effectors, supports the classification of the W10XG variants as T7SS-exported toxins.

To determine the frequency of association between PT-TG and W10XG proteins, we performed a secondary Pandoomain search using the W10XG HMM as a query. This analysis identified 62 W10XG-containing proteins (Table S5) lacking an associated PT-TG domain. Within this subset, 47 proteins were restricted to the genus *Priestia* and 37 exhibited high sequence conservation (>96% identity). These data indicate that while the W10XG domain is predominantly associated with PT-TG, this linkage is not universal and can be taxa-specific.

The discovery of the W10XG variant underscores the potential for false negatives in automated annotation pipelines. It also highlights the importance of manual curation and an understanding of the specific domains being studied to correctly interpret large data sets.

### Novel toxins discovery and validation

**Fig 7:**
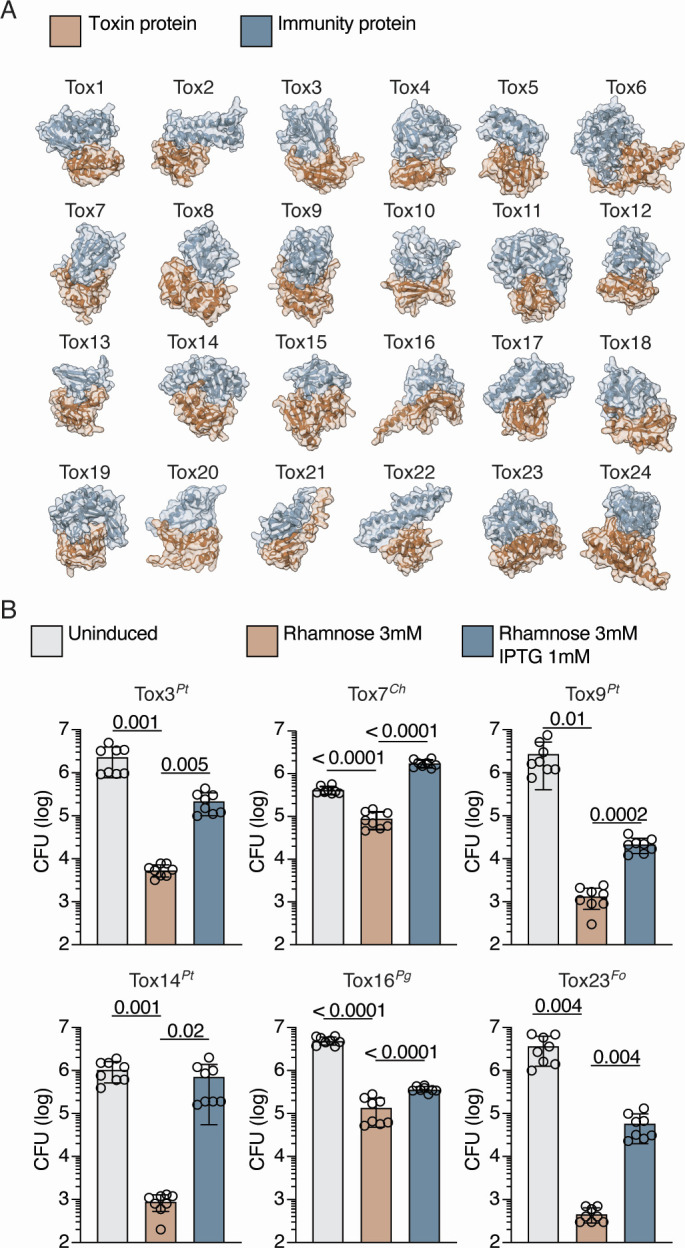
Structural diversity and experimental validation of novel W10XG-associated toxins. (**A**) AlphaFold3-predicted structural models of a representative toxin-immunity complex from each of the 24 novel toxin groups (Tox1–Tox24) discovered in association with the W10XG domain. The putative toxin is shown in brown and the cognate immunity protein in blue. Protein IDs for structural models are in Table S7. (**B**) Experimental validation of six representative toxin-immunity pairs. The bar charts show the viability of *E. coli* cells carrying inducible toxin and immunity protein expression plasmids. Each dot represents a biological replicate. Cultures were either left uninduced (control), induced for toxin expression (3 mM rhamnose), or induced for co-expression of the toxin and its immunity protein (3 mM rhamnose and 1 mM IPTG). Uppercase indicates the name of the species where the toxin and immunity protein were sourced: *Pt*, *Parageobacillus thermoglucosidasius*; *Ch*, *Caldifermentibacillus hisashii*; *Pg*, *Parageobacillus caldoxylosilyticus*; *Fo*, *Fredinandcohnia onubensis*. Statistical analysis was performed with Welch’s *t*-tests. The *P*-value is indicated above the bars.

We hypothesized that the absence of identifiable toxin domains in many W10XG proteins results from the presence of novel toxins that lack significant homology to established protein families. To identify these putative toxins, we implemented a filtering pipeline. Sequence alignment and AlphaFold3 structural modeling revealed a conserved ~260-residue N-terminal region comprising the W10XG and PT-TG domains, followed by a disordered linker. Based on these structural constraints, we removed proteins shorter than 260 residues to ensure inclusion of only candidates with a complete toxic domain. Next, we used Foldseek to search for structural homologs of known toxins. Finally, we refined this candidate list by selecting for proteins encoded immediately upstream of a putative immunity gene, a common genomic organization for toxin-immunity pairs. This criterion was further supported by structural modeling, which predicted a stable heterodimer interaction between the candidate toxin and its cognate immunity protein. This pipeline identified 68 putative toxins, which we subsequently clustered into 24 families based on high sequence and structural homology (Fig. 7A; Table S6).

To experimentally validate these predictions, we selected six representative toxin-immunity pairs for functional analysis. We synthesized codon-optimized versions of each toxin and immunity gene and cloned them into a dual-inducible *E. coli* expression system, with toxin expression controlled by rhamnose and immunity protein expression by IPTG. Upon induction, all six putative toxins caused a significant reduction in cell viability, ranging from one to three orders of magnitude. Co-expression of the cognate immunity protein reduced this toxic effect in all six pairs, resulting in a significant recovery of viable cells (Fig. 7B). The observed reduction in cell viability was a result of the specific bactericidal effect of toxins as the viability of cultures transformed with empty vectors or only immunity plasmids was not significantly reduced when compared to cultures before induction, with the exception of Imm7*^Ch^*, which showed a twofold reduction in growth but not bactericidal effect when the induced and uninduced cultures were compared (Fig. S2 and S3). These results provide strong experimental evidence that our pipeline successfully identified multiple groups of bona fide bacterial toxins.

The discovery of these novel toxin domains expands the known repertoire of interbacterial effectors. While Pandoomain-based annotations and structural modeling of the associated immunity proteins did not identify clear homologs with known cellular targets, the absence of signal peptides or transmembrane helices suggests a cytoplasmic localization. Consequently, we propose that these systems likely target nucleic acids or other cytosolic components rather than the cell envelope.

## DISCUSSION

The landscape of current bioinformatic tools for domain architecture and genomic context analysis creates a clear dichotomy between user-friendly web servers and high-throughput command-line pipelines, leaving a significant gap in accessible, end-to-end workflows. We created Pandoomain to specifically bridge this gap.

While web-based platforms such as the EFI-Genome Neighborhood Tool (19) and the InterPro (18) web interface are invaluable for visualizing specific gene neighborhoods, they lack the scalability needed for systematic, large-scale analysis. Conversely, command-line pipelines such as Bactopia (21), ProkFunFind (22), and Domainator (23) provide the computational resources needed for high-throughput exploration; they often lack an integrated, automated framework that seamlessly connects data acquisition from NCBI with downstream taxonomic and architectural processing.

Pandoomain provides a unified pipeline that automates the entire workflow from raw genome retrieval to the generation of comprehensive databases of protein domain information. Furthermore, by including the Pandoomain Browser, we link complex high-throughput analysis with intuitive user exploration, allowing researchers to visualize data locally in a web browser without advanced command-line proficiency. This integration allowed us to confirm our initial hypothesis that the PT-TG domain is part of a far broader range of antagonistic systems than previously recognized. By effectively automating the integration of taxonomic data alongside HMM-based domain identification and neighborhood analysis, Pandoomain provides the scalability required to uncover “false negative” domains, such as the novel W10XG variant, which are frequently missed by more rigid or less automated workflows.

Our analysis of the PT-TG domain provides validation of Pandoomain’s capacity for large-scale, context-driven discovery. By interrogating over 300,000 bacterial genomes, we identified 10,226 PT-TG-containing proteins organized into 312 unique domain architectures. This architectural diversity highlights the modularity of polymorphic toxin systems and confirms our hypothesis that the PT-TG domain serves as a versatile domain for both well-documented antagonistic systems, such as T6SS, T7SS, CDI, and novel protein modules, including bacterial Ig-like (Big) domains.

The integration of standardized taxonomic data was pivotal for interpreting these patterns. We observed distinct evolutionary trajectories, ranging from the restriction of choline-binding architectures to the *Bacillaceae* family, to the broad, phylum-spanning distribution of PT-TG singleton DA. These results demonstrate that PT-TG cellular function is likely independent of the cell envelope and thus is not involved in binding to the targeted cell or translocation. We hypothesize that the PT-TG function is to be a substrate for cleavage, allowing the release of the toxin domain.

Furthermore, our study reveals how Pandoomain overcomes the limitations of standard automated annotation, which often fails to identify novel trafficking domains due to low sequence homology. By leveraging genomic context analysis, we identified that proteins initially lacking recognizable domains frequently co-occurred with T7SS structural genes. This guilt-by-association led to the discovery of the W10XG domain variant, a finding that would likely remain masked in smaller-scale or purely sequence-based screens.

Beyond our analysis of the PT-TG domain, the modularity of the Pandoomain allows it to be readily adapted for diverse applications. For example, researchers with limited bioinformatic expertise are able to deploy Pandoomain to systematically identify novel conflict systems by analyzing genomic neighborhoods surrounding known immunity factors, or to scale the discovery of biosynthetic gene clusters across thousands of genomes, as performed in previous advanced *in silico* analysis (11–15). Ultimately, Pandoomain provides the scientific community with a scalable, automated framework to explore the “dark matter” of bacterial functions and their evolutionary dynamics.

## Supplementary Material

Reviewer comments

## Data Availability

The code required for Pandoomain implementation is available at https://github.com/deMoraes-Lab/Pandoomain. The files containing the genomes used in this study and their metadata, the annotation of proteins, and gene neighborhood information are publicly available in the open repository Zenodo (58).

## SUPPLEMENTAL MATERIAL

Supplemental figures msystems.00427-26-s0001.pdf Fig. S1-S3, supporting Fig. 6 and 7.

Table S1 msystems.00427-26-s0002.txt Protein architectures.

Table S2 msystems.00427-26-s0003.txt Domain single character codes.

Table S3 msystems.00427-26-s0004.xlsx Pfams from Fig. 4.

Table S4 msystems.00427-26-s0005.xlsx Results of Apriori test.

Table S5 msystems.00427-26-s0006.xlsx Protein ID of proteins containing W10XG and not PT-TG.

Table S6 msystems.00427-26-s0007.xlsx Protein IDs of proteins carrying W10XG and proteins in the newly identified toxin families.

Table S7 msystems.00427-26-s0008.xlsx The new toxin family and protein IDs.

OPEN PEER REVIEW reviewer-comments.pdf An accounting of the reviewer comments and feedback.

ASM does not own the copyrights to Supplemental Material that may be linked to, or accessed through, an article. The authors have granted ASM a non-exclusive, world-wide license to publish the Supplemental Material files. Please contact the corresponding author directly for reuse.
