# Supplementary material for: Pandoomain, a scalable pipeline for genomic and protein domain context analysis, reveals widespread PT-TG domain architectural diversity and novel polymorphic toxins
Source: mSystems. 2026 Jul 30;11(8):e00427-26. doi: 10.1128/msystems.00427-26 (PMC13483458; doi:10.1128/msystems.00427-26)
Supplement: Supplementary file 1 [file msystems.00427-26-s0001.pdf]

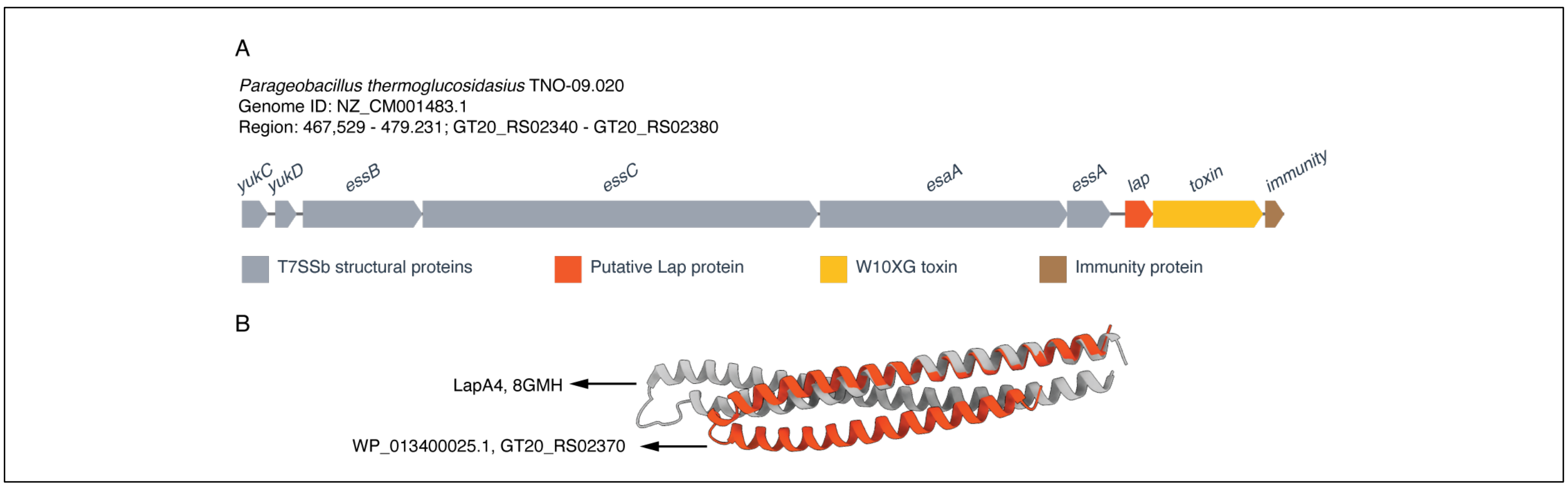

Figure S1. Genomic and structural evidence for the association of PT-TG toxins with Type VIIb secretion systems (T7SSb). (**A**) Representative genomic neighborhood of a PT-TG toxin. illustrating the co-occurrence of a PT-TG toxin containing a putative W10XG trafficking domain (yellow) with T7SSb structural components (grey), including yukC, yukD, essB, essC, esaA, and essA. A gene encoding a putative Lap-like protein (orange) is located immediately upstream of the toxin, followed by a downstream immunity protein (brown). (**B**) Structural homology of the identified Lap-like protein. Structural overlay of the predicted AlphaFold model for the *P. thermoglucosidasius* protein (WP_013400025.1; orange) with the known T7SSb chaperone LapA4 (PDB: 8GMH; grey).

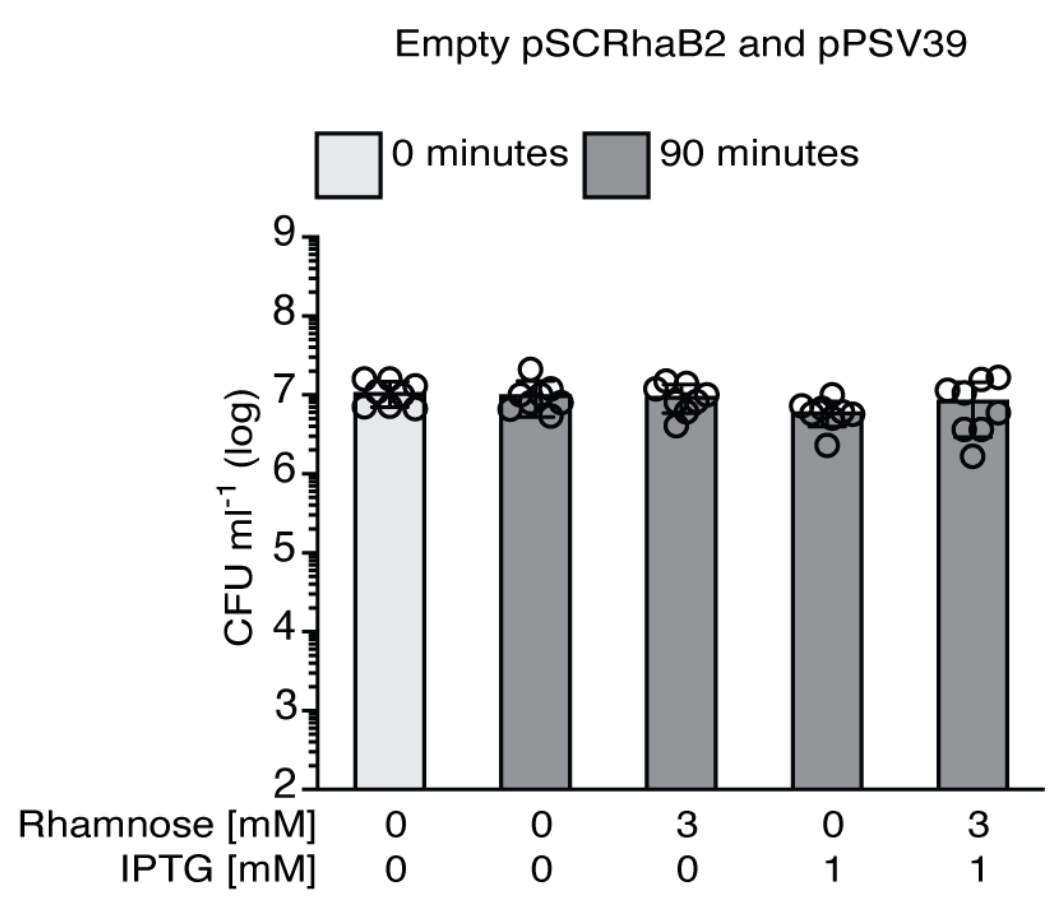

Fig S2. Growth of *E. coli* harboring empty expression vectors under inducing conditions. To ensure that the toxicity and rescue effects observed in Figure 7 were not artifacts of inducer concentrations or plasmid presence, colony-forming units (CFU ml^{-1}) were quantified for an *E. coli* strain carrying empty pSCRhaB2 and pPSV39 vectors. Cultures were sampled at 0 minutes (light grey) and 90 minutes (dark grey) post-induction under varying concentrations of rhamnose (0 to 3 mM) and IPTG (0 to 1 mM). Data points represent individual biological replicates, and error bars indicate the standard deviation of the mean. No significant differences in cell viability were observed across the varying concentrations of rhamnose and IPTG.

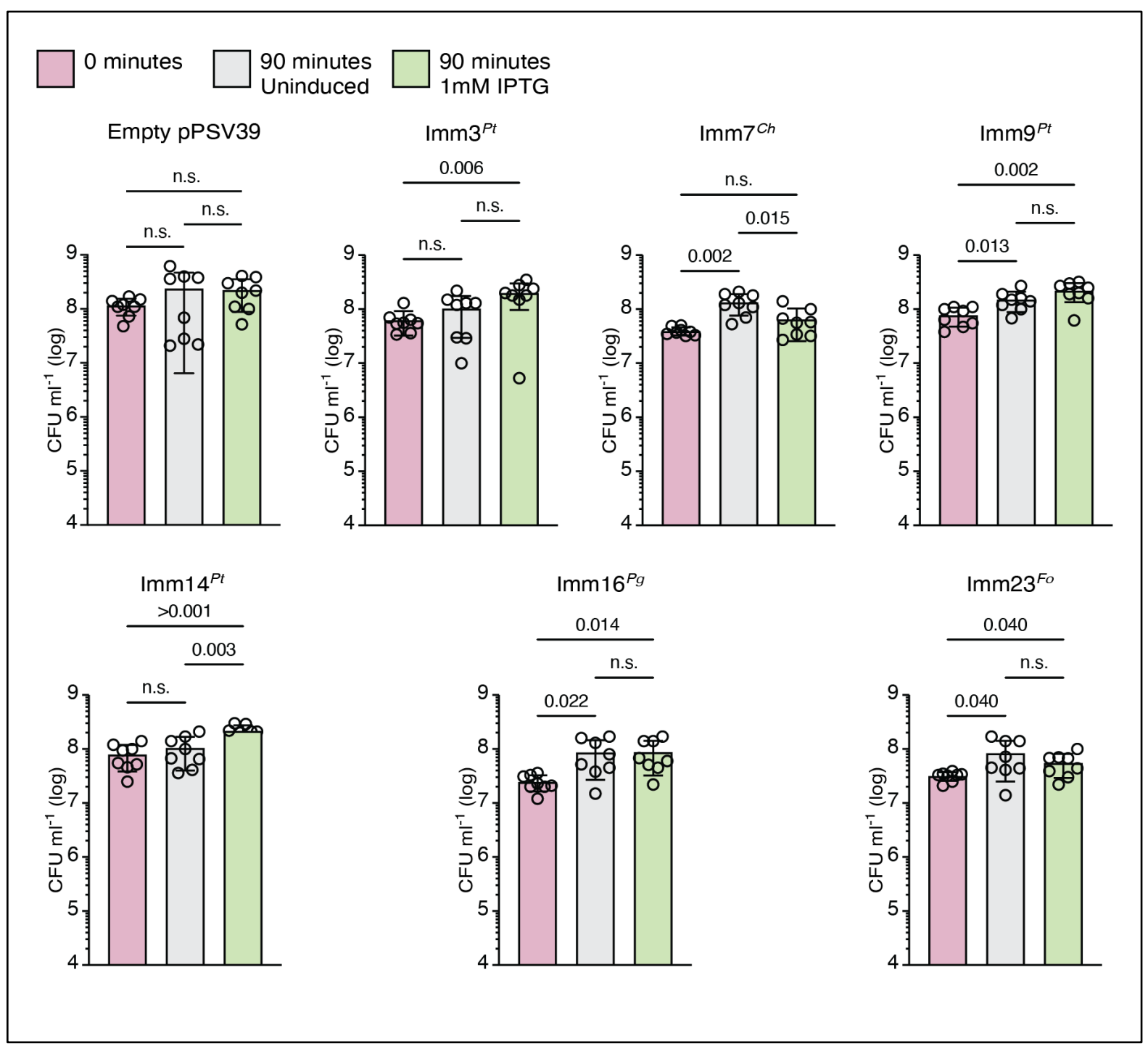

Fig S3. Growth of *E. coli* during independent expression of putative immunity proteins. To confirm that the candidate immunity proteins do not exhibit inherent toxicity, CFU counts were measured for *E. coli* strains carrying the indicated immunity genes in the pPSV39 vector. Cultures were sampled at 0 minutes (pink) and 110 minutes post-induction with 1 mM IPTG (green), alongside an uninduced control at 110 minutes (light grey). Uppercase indicates the name of the species where the toxin and immunity protein were sourced: *Pt* – *Parageobacillus thermoglucosidasius*, *Ch* – *Caldifermentibacillus hisashii*, *Pg* – *Parageobacillus caldoxylosilyticus*, *Fo* – *Fredinandcohnia onubensis*. Statistical analysis was performed with Welch's t-test. The p-value is indicated above the bars.
