# Supplementary material for: Pandoomain, a scalable pipeline for genomic and protein domain context analysis, reveals widespread PT-TG domain architectural diversity and novel polymorphic toxins
Source: mSystems. 2026 Jul 30;11(8):e00427-26. doi: 10.1128/msystems.00427-26 (PMC13483458; doi:10.1128/msystems.00427-26)
Supplement: Supplementary file 2 [file reviewer-comments.pdf]

Pandoomain, a scalable pipeline for genomic and protein domain context analysis, reveals widespread PT-TG domain architectural diversity and novel polymorphic toxins

Emanuel Soto, Adam Oliver, and Marcos de Moraes

Corresponding Author(s): Marcos de Moraes, Rice University

Review Timeline:

Submission Date:	May 5, 2026
Editorial Decision:	June 8, 2026
Revision Received:	June 26, 2026
Accepted:	July 8, 2026

Editor: Julia Willett

Reviewer(s): Disclosure of reviewer identity is with reference to reviewer comments included in decision letter(s). The following individuals involved in review of your submission have agreed to reveal their identity: Tracy Palmer (Reviewer #1); Jason E McDermott (Reviewer #3)

Transaction Report:

(Note: With the exception of the correction of typographical or spelling errors that could be a source of ambiguity, letters and reports are not edited. The original formatting of letters and referee reports may not be reflected in this compilation.)

DOI: https://doi.org/10.1128/msystems.00427-26

Re: mSystems00427-26 (Pandoomain, a scalable pipeline for genomic and protein domain context analysis, reveals widespread PT-TG domain architectural diversity and novel polymorphic toxins)

Dear Dr. Marcos H de Moraes:

Thank you for the privilege of reviewing your work. Below you will find my comments, instructions from the mSystems editorial office, and the reviewer comments.

Please address the comments from Reviewer 3, including the comment about revisions that were made to the introduction as these are not apparent from the Marked Up Manuscript file that was submitted.

Please return the manuscript within 60 days; if you cannot complete the modification within this time period, please contact me. If you do not wish to modify the manuscript and prefer to submit it to another journal, notify me immediately so that the manuscript may be formally withdrawn from consideration by mSystems.

Revision Guidelines
To submit your modified manuscript, log into the submission site at https://msystems.msubmit.net/cgi-bin/main.plex. Go to Author Tasks and click the appropriate manuscript title to begin. The information you entered when you first submitted the paper will be displayed; update this as necessary. Note the following requirements:

• Upload point-by-point responses to the issues raised by the reviewers in a file named "Response to Reviewers," NOT in your cover letter.
• Upload a compare copy of the manuscript (without figures) as a "Marked-Up Manuscript" file.
• Upload a clean .DOC/.DOCX version of the revised manuscript and remove the previous version.
• Each figure must be uploaded as a separate, editable, high-resolution file (TIFF or EPS preferred), and any multipanel figures must be assembled into one file.
• Any supplemental material intended for posting by ASM should be uploaded with their legends separate from the main manuscript. You can combine all supplemental material into one file (preferred) or split it into a maximum of 10 files with all associated legends included.

For complete guidelines on revision requirements, see our Submission and Review Process webpage. Submission of a paper that does not conform to guidelines may delay acceptance of your manuscript.

Data availability: ASM policy requires that data be available to the public upon online posting of the article, so please verify all links to sequence records, if present, and make sure that each number retrieves the full record of the data. If a new accession number is not linked or a link is broken, provide mSystems production staff with the correct URL for the record. If the accession numbers for new data are not publicly accessible before the expected online posting of the article, publication may be delayed; please contact production staff (mSystems@asmusa.org) immediately with the expected release date.

Publication Fees: For information on publication fees and which article types are subject to charges, visit ourwebsite. If your manuscript is accepted for publication and any fees apply, you will be contacted separately about payment during the production process; please follow the instructions in that e-mail. Arrangements for payment must be made before your article is published.

ASM Membership: Corresponding authors may join or renew ASM membership to obtain discounts on publication fees. Need to upgrade your membership level? Please contact Customer Service at Service@asmusa.org.

The ASM Journals program strives for constant improvement in our submission and publication process. Please tell us how we can improve your experience by taking this quick Author Survey.

Thank you for submitting your paper to mSystems.

Sincerely,
Julia Willett
Editor
mSystems

Reviewer #1 (Comments for the Author):

The authors have addressed all my concerns and the manuscript is much improved. Please note that track changes should be removed from the legend in Fig S3

Reviewer #3 (Comments for the Author):

I appreciate the responses from the authors to my comments and have the following additional comments:
1. Original comment #1: I agree that the work represents a significant advance. However, my question about use cases was not for my own knowledge, but rather a suggestion that some of these other approaches should be mentioned in the paper to let readers know what the possibilities might be. This could easily be incorporated in the discussion section, but you might also mention it in the introduction. As it stands the versatility (and potential uses) of Pandoomain are not easily apparent, and thus the application space may seem very limited (to the example case) to some readers - when it is actually a pretty powerful approach that may reveal other dark matter function. I apologize for not being more clear in my original comment.
2. Original comment #4: I had suggested some clarification of the take-home message for the paper. The authors provide a good response to the suggestion in their 'Responses' then stating that they 'Restructured the Introduction...' - however, I see very little change to the introduction, and nothing that indicates the suggestion I made was addressed here. The other portions of the response address my original comment, but if there are changes needed in the Introduction they should be made.

Reviewer #3 (Comments for the Author):

I appreciate the responses from the authors to my comments and have the following additional comments:

1. Original comment #1: I agree that the work represents a significant advance. However, my question about use cases was not for my own knowledge, but rather a suggestion that some of these other approaches should be mentioned in the paper to let readers know what the possibilities might be. This could easily be incorporated in the discussion section, but you might also mention it in the introduction. As it stands the versatility (and potential uses) of Pandoomain are not easily apparent, and thus the application space may seem very limited (to the example case) to some readers - when it is actually a pretty powerful approach that may reveal other dark matter function. I apologize for not being more clear in my original comment.

Response: We thank the reviewer for the clarification and agree that explicitly stating alternative use cases strengthens the manuscript by highlighting the pipeline's versatility. We have added text to the Introduction (#69-72) to outline broad applications and expanded the final paragraph of the Discussion (#456-463) to provide concrete examples of how researchers can adapt Pandoomain to uncover novel biology outside of polymorphic toxins.

2. Original comment #4: I had suggested some clarification of the take-home message for the paper. The authors provide a good response to the suggestion in their 'Responses' then stating that they 'Restructured the Introduction...' - however, I see very little change to the introduction, and nothing that indicates the suggestion I made was addressed here. The other portions of the response address my original comment, but if there are changes needed in the Introduction they should be made.

Response: We appreciate the reviewer highlighting the importance of clarifying the take-home message and the broad applicability of Pandoomain, as this improvement makes the paper stronger. To ensure the take-home message is immediately apparent, we have rewritten the final paragraph of the Introduction (#87-97).

Re: mSystems00427-26R1 (Pandoomain, a scalable pipeline for genomic and protein domain context analysis, reveals widespread PT-TG domain architectural diversity and novel polymorphic toxins)

Dear Dr. Marcos H de Moraes:

Your manuscript has been accepted, and I am forwarding it to the ASM production staff for publication. Your paper will first be checked to make sure all elements meet the technical requirements. ASM staff will contact you if anything needs to be revised before copyediting and production can begin. Otherwise, you will be notified when your proofs are ready to be viewed.

Data Availability: ASM policy requires that data be available to the public upon online posting of the article, so please verify all links to sequence records, if present, and make sure that each number retrieves the full record of the data. If a new accession number is not linked or a link is broken, provide production staff with the correct URL for the record. If the accession numbers for new data are not publicly accessible before the expected online posting of the article, publication may be delayed; please contact ASM production staff immediately with the expected release date.

Publication Fees: For information on publication fees and which article types have charges, please visit our website. We have partnered with Copyright Clearance Center (CCC) to collect author charges. If fees apply to your paper, you will receive a message from no-reply@copyright.com with further instructions. For questions related to paying charges through RightsLink, please contact CCC at ASM_Support@copyright.com or toll free at +1-877-622-5543. CCC makes every attempt to respond to all emails within 24 hours.

ASM Membership: Corresponding authors may join or renew ASM membership to obtain discounts on publication fees. Need to upgrade your membership level? Please contact Customer Service at Service@asmusa.org.

Embargo Policy: A press release may be issued as soon as the manuscript is posted on the mSystems Latest Articles webpage. The corresponding author will receive an email with the subject line "ASM Journals Author Services Notification" when the article is available online.

Cover Image Submissions: If you would like to submit a potential Cover Image, please email a file and a short legend to msystems@asmusa.org. Please note that we can only consider images that (i) the authors created or own and (ii) have not been previously published. By submitting, you agree that the image can be used under the same terms as the published article. Image File requirements: TIF/EPS, 7.5 inches wide by 8.25 inches tall (at least 2,250 pixels wide by 2,475 pixels tall), minimum 300 dpi resolution (600 dpi preferred), RGB, and no figure elements, e.g., arrows or panel labels. The legend should be a short description of the image, 1-2 sentences recommended. Please download and use this interactive template in Adobe to ensure that your proposed cover image meets our size requirements (https://journals.asm.org/pb-assets/pdf-text-excel-files/ASM-Interactive-Sizing-Cover-Template-1715689791.pdf).

Thank you for submitting your paper to mSystems.

Sincerely,
Julia Willett
Editor
mSystems
